# Exploring the roles and potential therapeutic strategies of inflammation and metabolism in the pathogenesis of vitiligo: a mendelian randomization and bioinformatics-based investigation

**DOI:** 10.3389/fgene.2024.1385339

**Published:** 2024-04-10

**Authors:** Ming-jie He, De-long Ran, Zhan-yi Zhang, De-shuang Fu, Qing He, Han-Yin Zhang, Yu Mao, Peng-Yuan Zhao, Guang-wen Yin, Jiang-an Zhang

**Affiliations:** ^1^ Department of Dermatology, First Affiliated Hospital of Zhengzhou University, Zhengzhou, Henan, China; ^2^ Department of Plastic and Reconstructive Surgery, The First Hospital of Jilin University, Changchun, Jilin, China

**Keywords:** vitiligo, inflammation, metabolism, mendelian randomization, bioinformatics, therapeutic strategies

## Abstract

**Introduction:**

Vitiligo, a common autoimmune acquired pigmentary skin disorder, poses challenges due to its unclear pathogenesis. Evidence suggests inflammation and metabolism’s pivotal roles in its onset and progression. This study aims to elucidate the causal relationships between vitiligo and inflammatory proteins, immune cells, and metabolites, exploring bidirectional associations and potential drug targets.

**Methods:**

Mendelian Randomization (MR) analysis encompassed 4,907 plasma proteins, 91 inflammatory proteins, 731 immune cell features, and 1400 metabolites. Bioinformatics analysis included Protein-Protein Interaction (PPI) network construction, Gene Ontology (GO), and Kyoto Encyclopedia of Genes and Genomes (KEGG) pathway analysis. Subnetwork discovery and hub protein identification utilized the Molecular Complex Detection (MCODE) plugin. Colocalization analysis and drug target exploration, including molecular docking validation, were performed.

**Results:**

MR analysis identified 49 proteins, 39 immune cell features, and 59 metabolites causally related to vitiligo. Bioinformatics analysis revealed significant involvement in PPI, GO enrichment, and KEGG pathways. Subnetwork analysis identified six central proteins, with Interferon Regulatory Factor 3 (IRF3) exhibiting strong colocalization evidence. Molecular docking validated Piceatannol’s binding to IRF3, indicating a stable interaction.

**Conclusion:**

This study comprehensively elucidates inflammation, immune response, and metabolism’s intricate involvement in vitiligo pathogenesis. Identified proteins and pathways offer potential therapeutic targets, with IRF3 emerging as a promising candidate. These findings deepen our understanding of vitiligo’s etiology, informing future research and drug development endeavors.

## 1 Introduction

Vitiligo is a common autoimmune acquired pigmentary skin disorder, affecting approximately 0.5%–2% of the global population (Eidsmo, 2022). Despite its prevalence, the exact etiology and pathogenesis of vitiligo remain elusive, involving a complex interplay of genetic predisposition and environmental triggers (Wang et al., 2021). While depigmented patches on the skin are the hallmark of vitiligo, the underlying mechanisms driving the disease extend beyond the visible symptoms. Recent advancements in research have illuminated the critical roles of inflammation and metabolic processes in the pathogenesis of vitiligo (Lyu and Sun, 2022).

Previous studies have confirmed abnormal inflammation and immune system activity to be associated with various autoimmune diseases (Needell and Zipris, 2017; Municio and Criado, 2021; Ruacho et al., 2022). As an autoimmune skin disorder, the pathogenesis of vitiligo has undergone extensive investigation, implicating widespread involvement of inflammatory factors and immune cells (Bergqvist and Ezzedine, 2021). Notably, interleukin (IL)-6, tumor necrosis factor (TNF)-α, IL-1β, interferon (IFN)-γ, IL-10, along with C-X-C motif chemokine ligand 10 (CXCL10), CXCL9, dendritic cells, natural killer cells, and CD8^+^ T cells, play pivotal roles in the onset and progression of vitiligo (Beyzaee et al., 2022; Hlača et al., 2022). Additionally, metabolic abnormalities have been linked to the pathogenesis of vitiligo, with some studies demonstrating significant differences in metabolic products in the blood of vitiligo patients compared to healthy controls (Tsoukalas et al., 2019; Ding et al., 2023).

However, past research has primarily consisted of observational studies, constrained by sample size and confounding factors, resulting in conflicting findings in some instances. For example, Laddha et al. (2012) reported elevated levels of TNFα in vitiligo patients compared to the control group, while several other studies have reached the opposite conclusion, finding no significant difference in TNFα concentration compared to normal controls (Pichler et al., 2009; Singh et al., 2012; Camara-Lemarroy and Salas-Alanis, 2013). Furthermore, although many observational studies have yielded relatively consistent results, such as increased expression of CXCL10 in vitiligo patients compared to the control group (Speeckaert et al., 2023), they often only provide correlational conclusions, making it challenging to establish causal relationships with vitiligo.

Mendelian randomization (MR) is a genetic epidemiological research method that utilizes single nucleotide polymorphisms (SNPs) as instrumental variables (IVs) (Burgess et al., 2019). It infers potential causal relationships based on Mendel’s laws of inheritance, offering several advantages over observational studies. Genetic variations are determined at conception, preceding disease development, and are generally not influenced by confounding factors such as postnatal factors and social environment. Therefore, causal relationships derived from MR studies exhibit more credible temporality, reducing confounding bias and minimizing the likelihood of reverse causation (Smith et al., 2007). This study employs a bidirectional two-sample MR research design, incorporating extensive datasets that encompass various biological factors, including inflammatory proteins, immune cell characteristics, and metabolites. Through bioinformatic analysis, we aim to elucidate the roles of the identified core proteins in cellular pathways and functions, providing potential targets for vitiligo treatment. Ultimately, through drug target exploration and molecular docking validation, we seek to propose potential therapeutic strategies based on biomarkers (Davies et al., 2018).

## 2 Materials and methods

### 2.1 Study design

**FIGURE 1:**
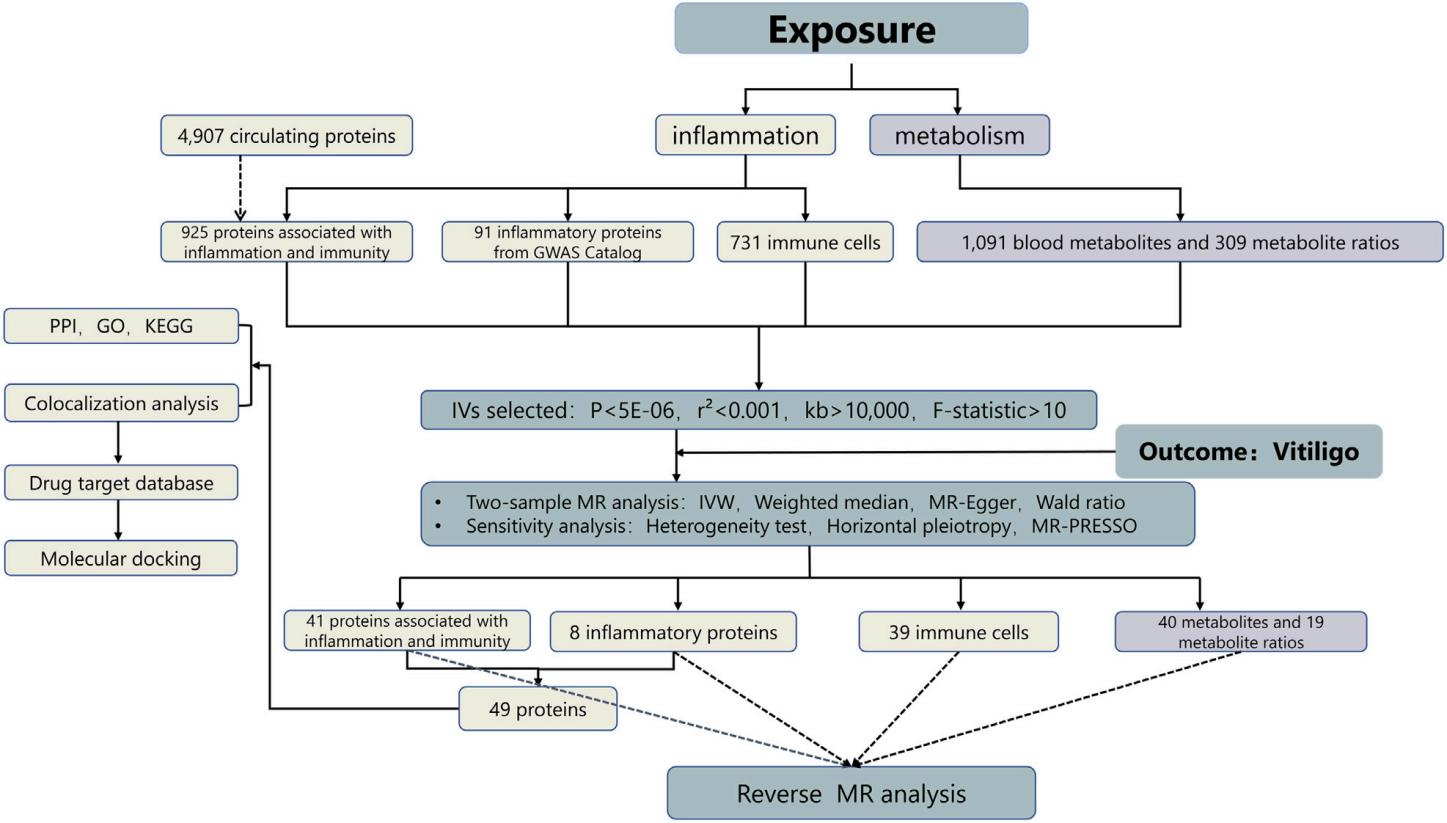
Study design. GO, gene ontology, IVs instrumental variables, KEGG, kyoto encyclopedia of genes and genomes, MR, mendelian randomization, PPI, protein–protein interaction.

To investigate the role of inflammation and metabolism in the pathogenesis of vitiligo, and to identify potential pharmacological targets and biomarkers, we employed a bidirectional two-sample MR analysis along with bioinformatics analysis, using primary data sourced from genome-wide association studies (GWAS) (Uitterlinden, 2016). Please refer to Figure 1 for detailed procedures.

### 2.2 Data sources

The data can be broadly categorized into exposure data and outcome data. The outcome data pertaining to vitiligo is sourced from the latest and most comprehensive Finnish database, R10 version (https://www.finngen.fi/en/access_results). Exposure data primarily consists of two major components: inflammation and metabolism. For metabolism analysis, we have incorporated 1,091 blood metabolites and 309 metabolite ratios obtained from the GWAS catalog (https:// www.ebi.ac.uk/gwas/studies/GCST90199621-902010209).Inflammation analysis is further subdivided into three components: 4,907 plasma proteins, 91 inflammatory proteins, and 731 immune cells. All GWAS data included in this study for MR analysis are of European ancestry. Refer to Supplementary Table S1 for detailed information regarding the data.

#### 2.2.1 Plasma protein screening

We utilized the circulating protein expression level GWAS study from deCODE Genetics (35,559 Icelanders, 4,907 proteins) to identify protein quantitative trait loci (pQTL). However, due to the presence of numerous proteins in plasma unrelated to inflammation and immunity, further filtering is necessary. We utilized the Gene Set Enrichment Analysis (GSEA) website (link: https://www.gsea-msigdb.org/gsea) to download human-relevant gene sets (H, C1-C8) from the Molecular Signatures Database. Subsequently, we filtered these gene sets using the keywords ‘inflammation’ and ‘immunity,’ resulting in 5,886 genes related to inflammation and immunity, as detailed in Supplementary Table S11. Next, we conducted an intersection operation between these genes and the 4,907 plasma proteins obtained from the deCODE dataset, yielding 925 proteins. Our focus was primarily on proteins associated with inflammation and immunity, thus completing the screening of plasma proteins.

#### 2.2.2 Merging with GWAS catalog for final protein selection

Merging the 925 proteins selected from Section 2.2.1, which are associated with inflammation and immunity, with the 91 inflammatory proteins from the GWAS catalog (https://www.phpc.cam.ac.uk/ceu/proteins), resulting in a final set of 1,016 proteins included in the MR analysis.

#### 2.2.3 Selection of immune cells

Data for 731 immune cell features are sourced from the GWAS catalog database (http://ftp.ebi.ac.uk/pub/databases/gwas/summary_statistics).

### 2.3 Genetic instrumental variable selection

In this section, we employed a rigorous process for the selection of genetic instrumental variables to ensure the robustness and reliability of our study. The steps involved in this selection are outlined below.

#### 2.3.1 Identification of SNPs significantly associated with the phenotype

We initially identified SNPs that exhibited a significant association with the phenotype, utilizing a stringent threshold (P < 5E-06) (He et al., 2024). All GWAS datasets included in this study provided *p*-values for the association between SNPs and exposure, similar to the *p*-value.exposure displayed in Supplementary Tables S2–S4.

#### 2.3.2 Removal of linkage disequilibrium (LD)

Removal of LD by applying quality control standards: r2 < 0.001, kb > 10,000 (Pierce et al., 2011). This step was implemented using the “clump_data” function within the “TwoSampleMR” package.

#### 2.3.3 Integration, concordance, and correction of palindromic SNPs

We integrated and assessed the concordance of the exposure-outcome dataset. Additionally, we corrected palindromic SNPs with ambiguous strands based on allele frequency information, ensuring accurate alignment and interpretation. This step was primarily implemented using the “harmonise_data” function within the “TwoSampleMR” R package. It automatically removes SNPs with palindromic sequences (e.g., where the effect allele is base C and the other allele is base G) during the final MR analysis.

#### 2.3.4 Assessment of instrumental variable (IV) strength

To evaluate the strength of the instrumental variables, we calculated the F-value. We excluded potentially weak IVs by setting a threshold (F > 10) to mitigate bias between the instrumental variables and exposure factors. The formula for calculating F is as follows: F=(R^2×(N-2))/(1-R^2), R^2=(2×β^2×EAF×(1-EAF))/(2×β^2×EAF×(1-EAF)+2×[SE]^2×N×EAF×(1-EAF)). Where (R^2) is the proportion of variation in the exposure database explained by SNPs, (N) represents the number of participants in the GWAS sample, (β) is the estimated effect size of the SNP, (SE) represents the standard error of the effect estimate, and (EAF) represents the effect allele frequency.

Refer to Supplementary Tables S2–S4 for detailed information regarding the SNP data.

### 2.4 MR analysis and sensitivity analysis

In this study, analysis was conducted using the “Two-Sample MR” and “MRPRESSO” packages in R 4.1.0 software. The primary method employed was the Inverse Variance Weighted (IVW) method to calculate the odds ratio (OR) and its 95% confidence interval (CI), assessing the potential causal relationship between exposure and outcome. Additionally, supplementary analyses were performed using MR-Egger regression and Weighted Median Method (WME), with the Wald Ratio method applied for exposures with only one SNP (Bowden et al., 2015; Bowden et al., 2016; Perry et al., 2021). Subsequently, sensitivity analyses were conducted to ensure the validity and robustness of the MR analysis results. For heterogeneity assessment, Cochran’s Q was employed to test SNP heterogeneity. If *p* < 0.05, indicating heterogeneity, a random-effects model was used; otherwise, a fixed-effects model was applied. To assess horizontal pleiotropy, MR-Egger method and MRPRESSO (MR pleiotropy residual and outlier) method were jointly utilized. Exposure data exhibiting horizontal pleiotropy were removed to ensure the reliability of conclusions. To address the issue of multiple testing, the Benjamini–Hochberg method was employed, which incorporates the false discovery rate (FDR). The significance threshold was set at *p* < 0.05. The exposure with both original *p* values and FDR-corrected *p* values less than 0.05 is considered to have a significant causal relationship with vitiligo, while the exposure with an original *p*-value less than 0.05 but an FDR-corrected *p*-value greater than 0.05 is considered to have a potential causal relationship with vitiligo.

### 2.5 Bioinformatics analysis

Following the outlined procedures, we identified a total of 49 inflammation-immune-related proteins causally associated with vitiligo. Subsequent bioinformatics analysis was executed on this protein set.

#### 2.5.1 Protein-Protein Interaction (PPI) network construction

Utilizing the STRING database (https://string-db.org/), we retrieved and validated the aforementioned 49 inflammation-immune-related proteins. Leveraging known physical interactions and functional relationships, we constructed a comprehensive Protein-Protein Interaction (PPI) network (Szklarczyk et al., 2023).

#### 2.5.2 GO and KEGG analysis

Conducting Gene Ontology (GO) functional enrichment analysis and Kyoto Encyclopedia of Genes and Genomes (KEGG) pathway analysis on the 49 proteins offered additional insights into their roles across biological processes (BP), cellular components (CC), molecular functions (MF), and pathways.

#### 2.5.3 Subnetwork discovery and identification of hub proteins

To unveil functional modules and hub regulatory proteins within the PPI network, we employed the Molecular Complex Detection (MCODE) plugin in Cytoscape software for subnetwork discovery. We set parameters (degree cutoff = 2, node score cutoff = 0.2, k-core = 2, and max. depth = 100) for optimal results (Menon and Elengoe, 2020).

#### 2.5.4 Colocalization analysis

For the six hub proteins identified by MCODE, we performed co-localization analysis using the R package coloc (Wallace, 2021). Bayesian co-localization assesses the probability that a protein and vitiligo share the same SNP, mitigating bias introduced by linkage disequilibrium (LD) in MR analysis (Giambartolomei et al., 2014). In co-localization analysis, five hypotheses were considered.

(1) H0: Unrelated to both vitiligo and the protein (PP0).
(2) H1: Related to the protein, unrelated to vitiligo (PP1).
(3) H2: Related to vitiligo, unrelated to the protein (PP2).
(4) H3: Related to either the protein or vitiligo, but with independent SNPs (PP3).
(5) H4: Related to both the protein and vitiligo, with shared SNPs (PP4).

Particular attention was given to the H4 hypothesis, and when PP.H4 exceeded 0.75, it was considered strong evidence of co-localization.

#### 2.5.5 Exploration of drug targets and molecular docking validation

Through successive filtering, we identified hub proteins with promising drug target potential. Records of past or ongoing clinical drug development projects for these proteins were retrieved from the Therapeutic Target Database (http://db.idrblab.net/ttd/) and ClinicalTrials.gov (https://clinicaltrials.gov/). To assess the binding affinity and interaction patterns between the candidate drug/small molecule and its target, molecular docking validation was conducted using Autodock software (Morris et al., 2008). Two-dimensional protein structures were obtained from the Protein Data Bank (PDB) (https://www.rcsb.org/), and the chemical structures of drugs were searched on PubChem (https://pubchem.ncbi.nlm.nih.gov/) (Wang et al., 2017).

## 3 results

### 3.1 Results of MR analysis and sensitivity analysis

**FIGURE 2:**
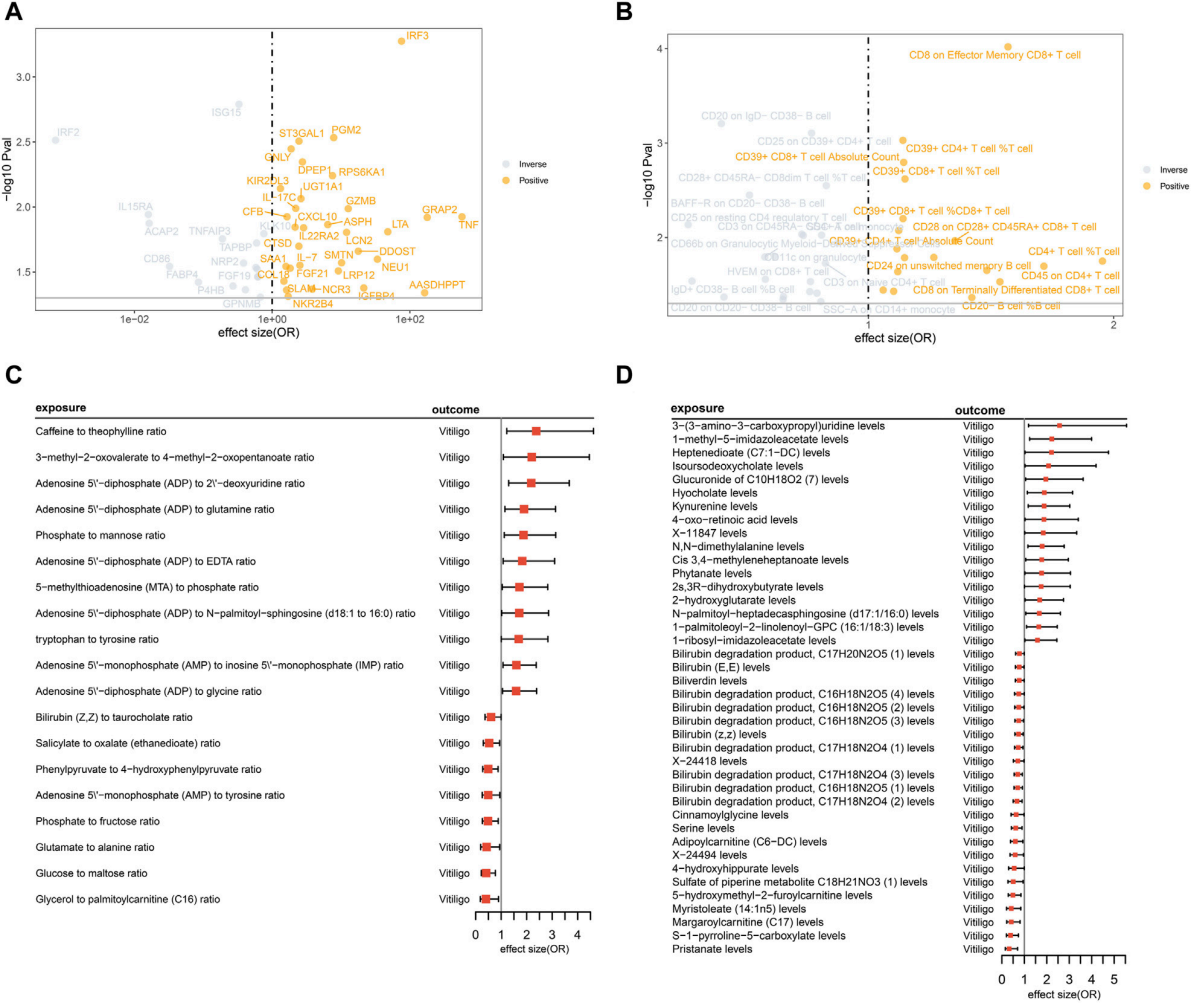
MR analysis illustrates the causal relationships between inflammatory-immune-related proteins, immune cells, metabolites, and metabolite ratios with vitiligo. **(A)** The volcano plot displays the causal relationships between 49 inflammatory-immune-related proteins and vitiligo. However, certain protein names, including TNFRSF11B, TNFSF12, SELL, TLR3 (all with odds ratios less than 1), were not displayed due to overlapping positions, represented by gray circles; **(B)** The volcano plot displays the causal relationships between 39 immune cell features and vitiligo; **(C)** The forest plot presents the causal relationships between 19 metabolite ratios and vitiligo; **(D)** The forest plot presents the causal relationships between 40 metabolites and vitiligo.

Through conducting MR analysis on multiple plasma proteins, inflammatory proteins, immune cell features, and metabolites related to inflammation, immunity, and metabolism, we have identified a series of biomarkers causally associated with vitiligo. Detailed results can be found in Figure 2; Supplementary Tables S5–S7. Sensitivity analysis results can be found in Supplementary Tables S8–S10.

#### 3.1.1 Proteins

From the initial screening of 925 inflammation-immune-related proteins and 91 inflammatory proteins, a total of 49 proteins causally related to vitiligo were identified through MR analysis. No horizontal pleiotropy was observed among these proteins. After FDR correction, the *p* values of six proteins remain less than 0.05: IRF2, IRF3, ISG15, PGM2, ST3GAL1, GNLY.

#### 3.1.2 Immune cell

Through MR analysis of 731 immune cell features, we identified 45 immune cell features causally associated with vitiligo.

Due to the presence of horizontal pleiotropy in six features, they were excluded from the final results, resulting in 39 immune cell features. After FDR correction, the *p* values of eight immune cell phenotypes remain less than 0.05: CD8 on Effector Memory CD8^+^ T cell, CD20 on IgD- CD38^−^ B cell, CD25 on CD39^+^ CD4^+^ T cell, CD39^+^ CD4^+^ T cell %T cell, CD39^+^ CD8^+^ T cell Absolute Count, CD39^+^ CD8^+^ T cell %T cell, CD28^+^ CD45RA- CD8dim T cell %T cell, and BAFF-R on CD20^−^ CD38^−^ B cell.

#### 3.1.3 Metabolites and metabolite ratios

MR analysis of 1,400 metabolites and metabolite ratios revealed 61 causal relationships with vitiligo.

Two were excluded due to horizontal pleiotropy, resulting in a final set of 59 metabolites and metabolite ratios. After FDR correction, the *p* values of 59 metabolites and metabolite ratios are all greater than 0.05, suggesting potential causal relationships with vitiligo.

### 3.2 Reverse MR analysis

**FIGURE 3:**
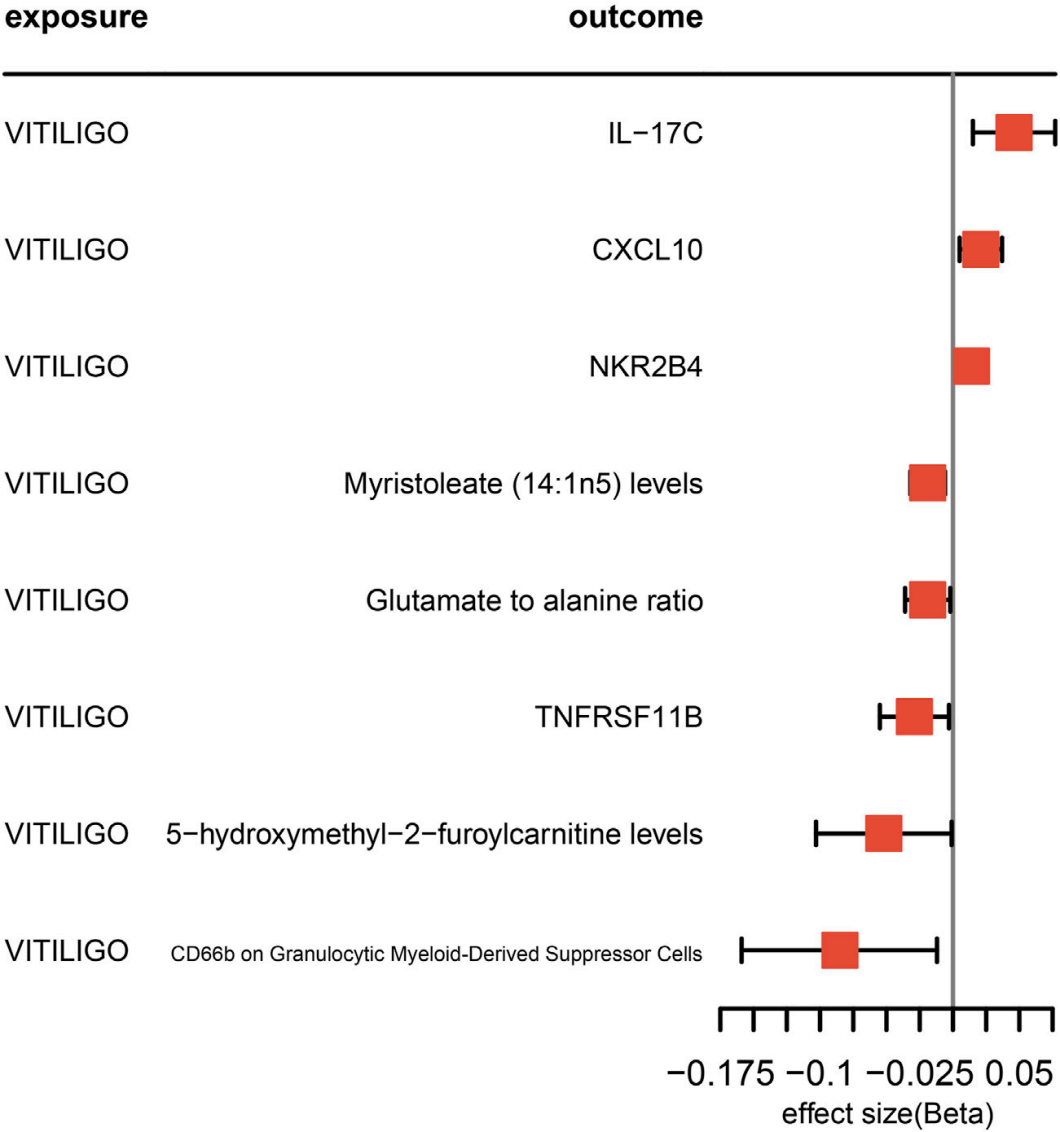
The forest plot illustrates the results of the reverse Mendelian Randomization analysis when vitiligo is considered as the exposure.

Using vitiligo as the exposure and the aforementioned 49 proteins, 39 immune cell features, and 59 metabolites and ratios as outcomes, we analyzed for bidirectional associations. The results revealed four proteins, three metabolites and ratios, and one immune cell phenotype exhibiting bidirectional causal relationships. Detailed results can be found in Figure 3.

### 3.3 Bioinformatics analysis

#### 3.3.1 PPI analysis results

**FIGURE 4:**
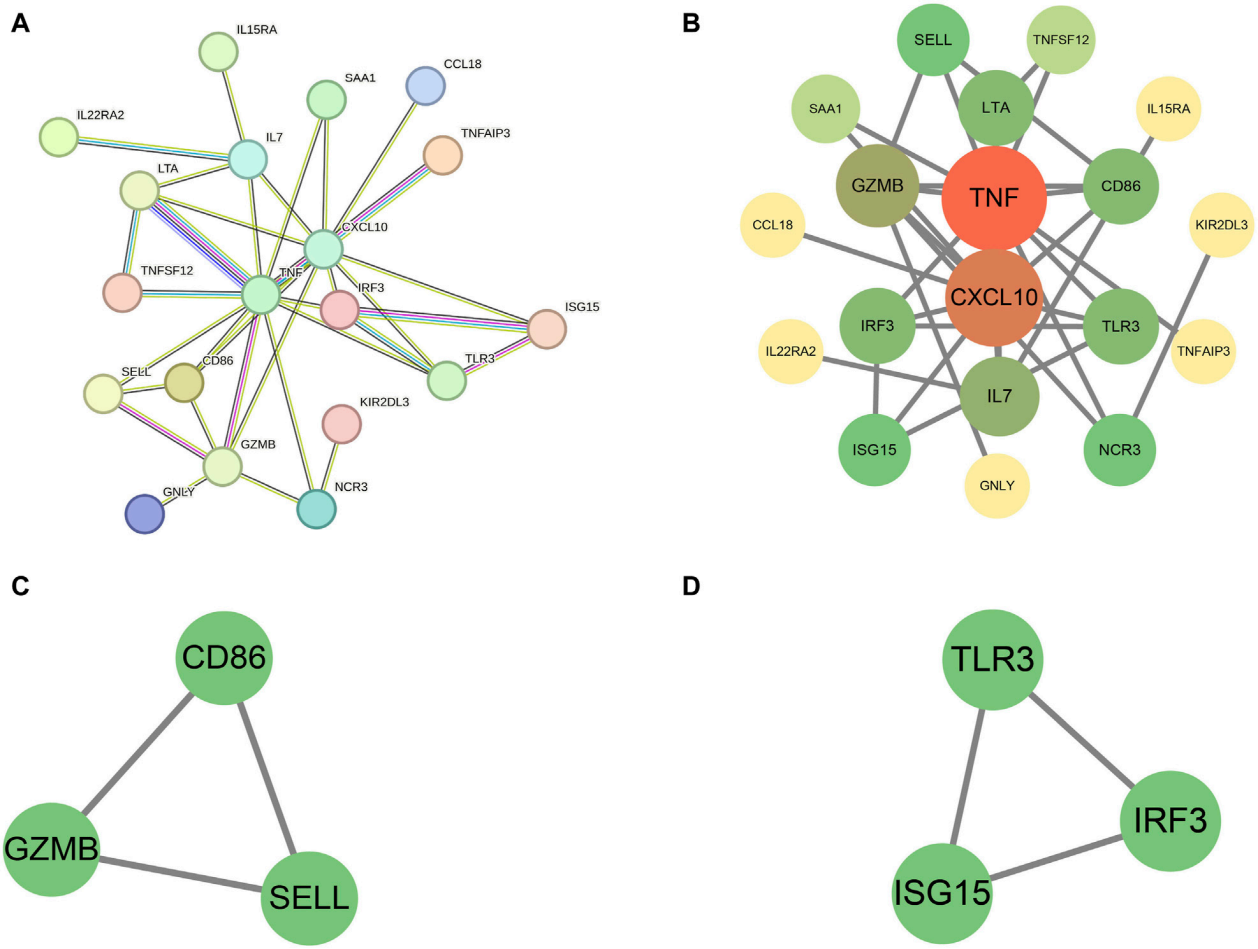
**(A)** The PPI graph created using the STRING website; **(B)** Further processing of the 19 proteins using Cytoscape software; **(C,D)** Identification of two subnetworks comprising six hub proteins using the MCODE plugin.

We subjected the 49 proteins to Protein-Protein Interaction (PPI) analysis using the STRING website, with a minimum required interaction score set to high confidence (0.700). Under this criterion, we identified interactions among 19 proteins, and these relationships are detailed in Figures 4A, B. Notably, TNF and CXCL10 had the highest number of connections with other proteins.

#### 3.3.2 GO and KEGG analysis

**FIGURE 5:**
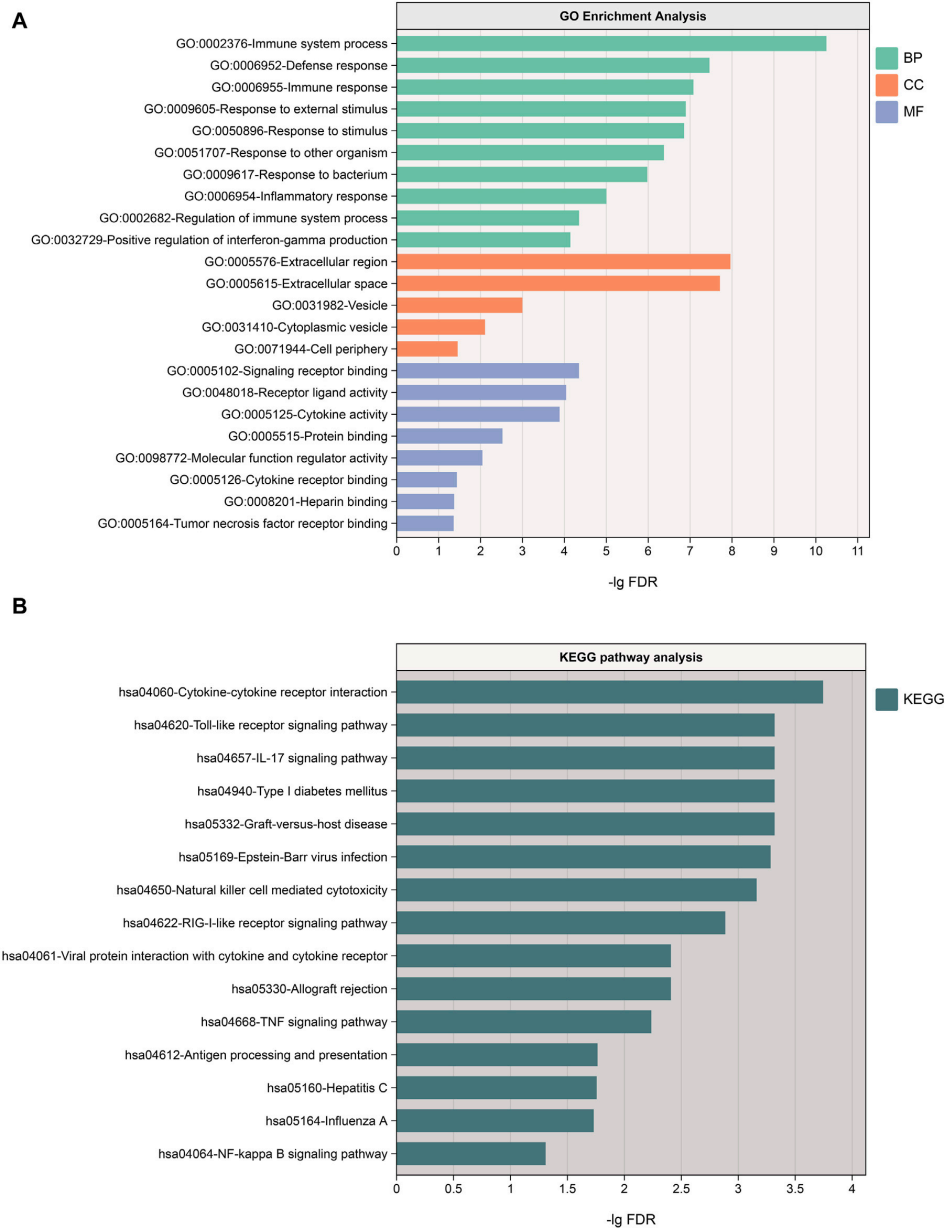
**(A)** Results of GO enrichment analysis, sorted by FDR values, with only the top 10 BP pathways displayed; **(B)** Results of KEGG pathway analysis. BP, biological processes; CC, cellular components, MF, molecular functions.

We conducted GO and KEGG analyses on the 49 proteins through the STRING website. The results revealed the most significant BP as the immune system process, CC primarily located in the extracellular region, and MF involving signaling receptor binding. Additionally, KEGG pathway analysis highlighted the most significant pathway as cytokine-cytokine receptor interaction. These findings collectively underscore the importance of these proteins in the immune system. For a more detailed analysis, please refer to Figure 5.

#### 3.3.3 Results of subnetwork discovery and identification of hub proteins

Utilizing Cytoscape’s MCODE plugin, we identified two subnetworks comprising six hub proteins: CD86, granzyme B (GZMB), selectin L (SELL), toll like receptor 3 (TLR3), interferon regulatory factor 3 (IRF3), and ISG15. These proteins may play more pivotal regulatory roles. For further details, please refer to Figures 4C, D.

#### 3.3.4 Results of colocalization analysis

**FIGURE 6:**
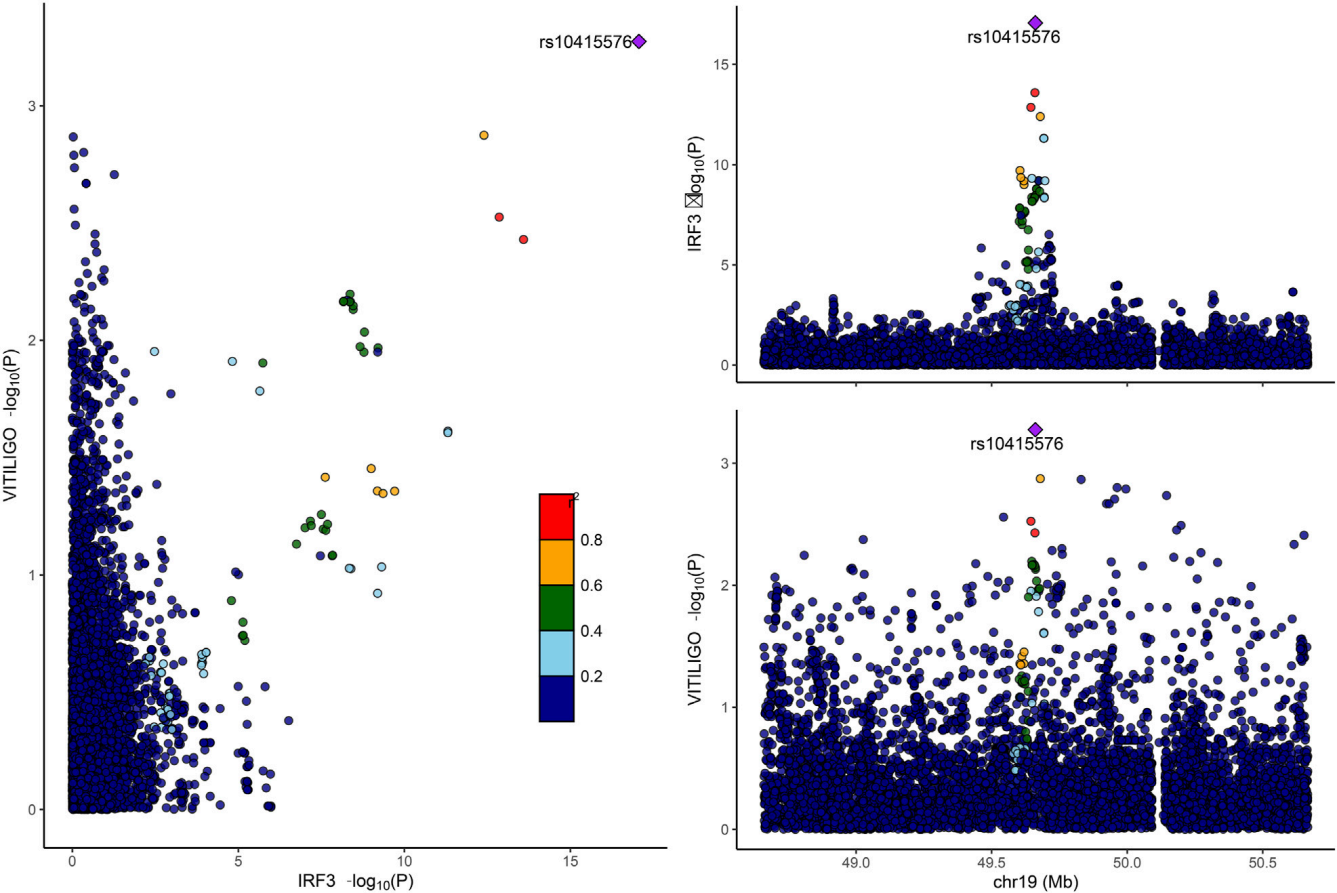
Displays the colocalization analysis results between IRF3 and vitiligo. IRF3, interferon regulatory factor 3.

Only IRF3 among the six hub proteins passed the co-localization analysis (PP.H4 > 0.75). Detailed results can be found in Figure 6. However, it is noteworthy that a negative co-localization result does not necessarily imply the ineffectiveness of the MR analysis.

#### 3.3.5 Results of exploration of drug targets and molecular docking validation

**FIGURE 7:**
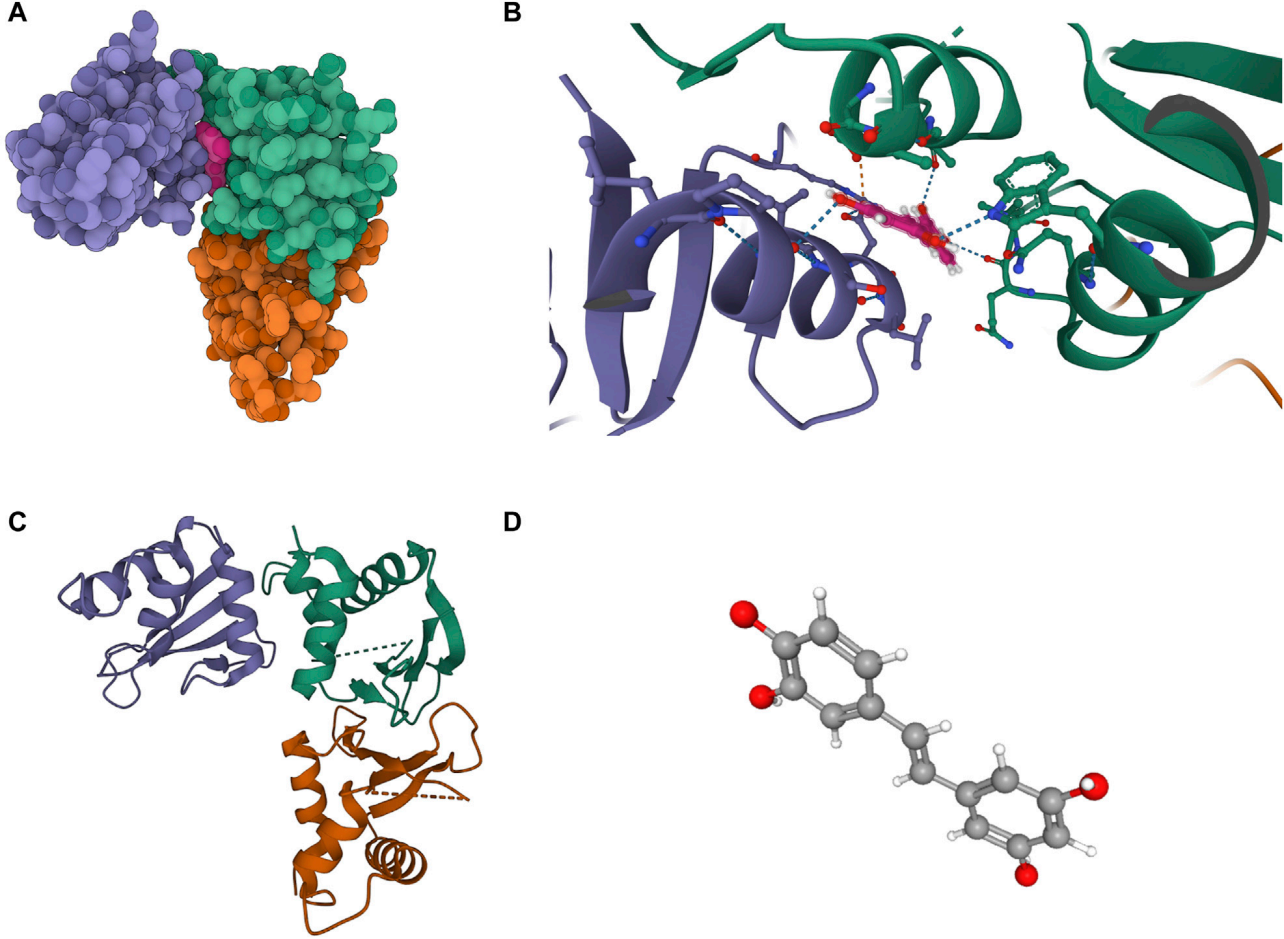
Binding mode of screened drugs to their targets by molecular docking. **(A)** Cartoon representation, overlay of the crystal structures of small molecule compounds and their targets were illustrated by Molecule of the Month feature; **(B)** The PyMOL software displays the three-dimensional structure of the binding pocket along with the linkage between the compound and its target; **(C)** The 3D structure of IRF3; **(D)** The 3D structure of Piceatannol.

The molecular structure of Piceatannol (Compound CID: 667,639) was obtained from the PubChem compound database (https://pubchem.ncbi.nlm.nih.gov/). The 3D coordinates of the protein IRF3 (PDB code: 3QU6; resolution: 2.3 Å) were downloaded from the Protein Data Bank (PDB) (http://www.rcsb.org/). Molecular docking results indicate that Piceatannol binds to IRF3 through visible hydrogen bonds and strong electrostatic interactions. Piceatannol successfully occupies the hydrophobic pocket of IRF3. The binding energy is −7.293 kcal/mol, suggesting a highly stable binding. Detailed results can be found in Figure 7.

## 4 Discussion

In this study, through MR analysis, we validated the causal relationships between 49 inflammation-immune-related proteins and vitiligo. Notably, proteins such as IL-17C, CXCL10, NKR2B4 (CD244), and TNF receptor superfamily member 11b (TNFRSF11B) exhibited bidirectional causality with vitiligo. GO enrichment analysis unveiled the involvement of these proteins in multiple biological processes, including inflammatory responses, immune regulation, and positive regulation of interferon-gamma production. Additionally, they were associated with cellular components such as extracellular region and vesicles, as well as molecular functions like receptor binding, receptor ligand activity, and cytokine receptor binding. KEGG analysis further underscored the significance of the Cytokine-cytokine receptor interaction pathway. PPI analysis revealed the interplay among these proteins, with TNF and CXCL10 showing the highest connectivity. The MCODE plugin identified six hub proteins, including CD86, GZMB, SELL, TLR3, IRF3, and ISG15. IRF3, supported by co-localization analysis, was associated with vitiligo and holds potential as a therapeutic target. Drug target exploration suggested that the small molecule Piceatannol could serve as an inhibitor for IRF3, and molecular docking validated the stable affinity between them. Further research is required to ascertain whether Piceatannol can effectively treat vitiligo by inhibiting IRF3. After conducting MR analysis on 1,400 metabolites and metabolite ratios, we confirmed 40 metabolites and 19 metabolite ratios causally linked to vitiligo, including 11 related to Bilirubin and its metabolites, suggesting a potential protective role against vitiligo. Zhang et al. (2018) revealed a significant decrease in serum Heme Oxygenase-1 (HO-1) and its metabolites, including Bilirubin, CoHb, and iron concentrations in vitiligo patients compared to the healthy control group. They successfully controlled the progression of vitiligo by using an HO-1 agonist to restore the functionality of regulatory T cells (Tregs). This finding suggests that HO-1 might be a potential therapeutic target for vitiligo. Based on our research results, we speculate that the protective effect of HO-1 on vitiligo is likely closely associated with Bilirubin. Additionally, we identified causal relationships between 731 immune cell features and vitiligo. We confirmed 39 immune cell features causally linked to vitiligo, with CD8 on Effector Memory CD8^+^ T cells showing the highest significance (*p* = 9.58E-05). In addition to CD8^+^ T cells, we should also pay attention to other immune cells that may potentially have a protective effect against vitiligo. For instance, the presence of CD66b on Granulocytic Myeloid-Derived Suppressor Cells (*p* = 1.61E-02) has drawn our attention. This is consistent with the findings of Douguet et al. (2018), who utilized a transgenic mouse model carrying the ret oncogene (Ret mice) that develops a spontaneous metastatic melanoma and observed a reduction in the number of Myeloid-Derived Suppressor Cells (MDSCs) at the primary tumor site in mice with vitiligo. This suggests that MDSCs may play a protective role in the development of vitiligo to some extent. It is intriguing to note the close associations between MDSCs and various inflammatory proteins and metabolites identified in our study. For instance, Tran et al. (2020) found that Bilirubin enhances the recruitment of MDSCs and suppresses the activities and functions of T cells in blood in the sepsis mouse model. According to Lu et al. (2021), upregulation of CXCL10 in a murine renal cancer model was associated with a reduction in the frequency and immunosuppressive activity of MDSCs. Additionally, Cheng et al.'s research (Cheng et al., 2020) indicated that cGAMP, by stimulating the cGAS-cGAMP-STING-IRF3 pathway, decreased the quantity of MDSCs, suggesting a potential inhibitory role of IRF3 in regulating MDSCs numbers. Interestingly, our findings suggest a protective role of both MDSCs and Bilirubin against vitiligo, while IRF3 and CXCL10 may potentially increase the risk of vitiligo occurrence.

Current research suggests that vitiligo results from the combined effects of genetic factors (approximately 80%) and environmental stressors (about 20%) (Bergqvist and Ezzedine, 2021). Under this interplay, melanocytes in vitiligo patients are more susceptible to oxidative stress, leading to cellular damage (Jadeja et al., 2020; Chang and Ko, 2023). This process prompts melanocytes to release exosomes containing specific antigens, activating CD8^+^ T cells to produce various cytokines such as IFNγ, TNF, and GZMB. Notably, IFNγ induces the secretion of CXCL9 and CXCL10 by keratinocytes, where CXCL10, through interaction with CXCR3B, induces apoptosis of melanocytes (Tulic et al., 2019; Su et al., 2020; Bergqvist and Ezzedine, 2021). Our research findings robustly confirm a bidirectional positive causal relationship between CXCL10 and vitiligo. On one hand, the increase in CXCL10 contributes to the development of vitiligo, and on the other hand, the presence of vitiligo leads to a significant upregulation of CXCL10 expression. This discovery aligns with previous studies, emphasizing CXCL10 as a potential effective target for treating vitiligo. Our study also addresses some controversies in previous research, confirming a causal relationship between TNF and vitiligo, indirectly supporting the rationale for using TNF inhibitors in vitiligo treatment (Kemp, 2015). However, we also note that some tumor necrosis factor inhibitors may induce the onset of vitiligo, a phenomenon observed in patients with various other conditions, such as hidradenitis suppurativa, ankylosing spondylitis, Crohn’s disease, and psoriasis (Dunn et al., 2019; Anthony et al., 2020; Phan et al., 2020). This paradoxical result prompts further consideration. Interestingly, through MR analysis, we identified TNFRSF11B, TNF alpha induced protein 3 (TNFAIP3), TNF superfamily member 12 (TNFSF12) as potentially protective factors against vitiligo, which may explain why some patients experience depigmentation after using TNF inhibitors.

In our study, we made a notable discovery, revealing for the first time the potential involvement of IRF3 in the pathogenic mechanism of vitiligo. Previous research by Sen et al. (2019) indicated that the inhibition of DNA damage repair proteins poly ADP-ribose polymerase (PARP) and checkpoint kinase 1 (CHK1) significantly increases PD-L1 expression in patients with small cell lung cancer (SCLC), thereby activating the STING/TBK1/IRF3 immune pathway. Activation of this pathway leads to the release of chemokines such as CXCL10, inducing the activation of cytotoxic T lymphocytes. We hypothesize that in vitiligo, IRF3 might contribute to the development of the condition by promoting the release of CXCL10. Furthermore, findings from the study by Dang et al. (2004) further support the importance of IRF3 in immune regulation. Their experiments in a mouse model of septic shock revealed that Piceatannol exhibits inhibitory effects by effectively blocking lipopolysaccharide (LPS)-mediated IRF3 activation. This inhibitory effect, achieved by downregulating the expression of various inflammatory factors, successfully suppressed the occurrence of inflammation. These results provide additional support to our discovery, suggesting that IRF3 may serve as a crucial node in the regulation of inflammation. Piceatannol, acting as an inhibitor of IRF3, may play a role in modulating the pathogenic mechanism of vitiligo. Although our molecular docking validation demonstrated the affinity between Piceatannol and IRF3, further in-depth research and validation are necessary to explore the therapeutic potential of Piceatannol in vitiligo.

Our research has certain limitations that need to be acknowledged. Firstly, we focused solely on the causal relationships between peripheral blood protein levels, immune cells, metabolites, and vitiligo, without considering skin tissue. This limitation arises from the unavailability of large, publicly accessible GWAS datasets specifically related to skin tissue. Secondly, our study exclusively covers the European population, potentially restricting the generalizability of conclusions to other ethnic groups.

In summary, our study revealed causal relationships between 49 proteins, 39 immune cell features, and 59 metabolites with vitiligo. We addressed some controversies present in traditional observational studies and conducted in-depth exploration. Notably, we identified IRF3 as a potential novel therapeutic target for vitiligo. These research findings provide crucial insights for a deeper understanding of the pathogenic mechanisms of vitiligo and the development of future therapeutic strategies.

## Data Availability

The original contributions presented in the study are included in the article/Supplementary Material, further inquiries can be directed to the corresponding authors.
